# Löffler endocarditis: a rare cause of acute cardiac failure

**DOI:** 10.1186/1749-8090-7-109

**Published:** 2012-10-10

**Authors:** Nicolasine D Niemeijer, Paul LA van Daele, Kadir Caliskan, Frans BS Oei, Olaf JL Loosveld, Nardo JM van der Meer

**Affiliations:** 1 Department of Internal Medicine, Erasmus Medical Centre, Rotterdam, the Netherlands, P.O. Box 2040, , 3000, CA, Rotterdam, the Netherlands; 2 Department of Internal Medicine, Amphia Hospital, Breda, the Netherlands; 3 Department of Cardiology, Erasmus Medical Centre, Rotterdam, the Netherlands; 4 Department of Cardio-Thoracic Surgery, Erasmus Medical Centre, Rotterdam, the Netherlands; 5 Department of Anaesthesiology and Intensive Care Medicine, Amphia Hospital, Breda and Oosterhout, the Netherlands

**Keywords:** Hypereosinophilic syndrome, Cardiac thrombus, Cardiac failure, Löffler endocarditis

## Abstract

We describe a patient with acute cardiogenic shock due to cardiac involvement in idiopathic hypereosinophilic syndrome (Löffler endocarditis). At the echocardiography, there was a huge mass in the left ventricular cavity, resulting in inflow- and outflow tract obstruction. The posterior leaflet of the mitral valve apparatus was completely embedded in a big (organized) thrombus mass. The patient was treated with high dose corticosteroids, however without effect. Partial remission was achieved after treatment with hydroxycarbamide. He was also treated with anticoagulants and high dose beta-blockers. The patient’s condition improved remarkably after correction of the mitral valve insufficiency by a mitral valve bioprosthesis.

## Background

The hypereosinophilic syndromes (HES) are a heterogenous group of disorders marked by the sustained overproduction of eosinophils. It is a rare condition with an unknown prevalence. Diagnosis is based on three criteria: (1) an eosinophil count of more than 1500 cells per microliter for at least 6 months, (2) no other evident cause for eosinophilia, including allergic diseases and parasitic infection and (3) sign or symptoms of organ involvement by eosinophilic infiltration [1]. Cardiac involvement is common in HES and eosinophilic myocarditis is a major cause of morbidity and mortality among patients with HES. Mitral valve involvement may lead to acute cardiac failure. We present a patient with idiopathic hypereosinophilic syndrome and severe cardiac involvement, so-called Löffler endocarditis.

## Case report

A 37-year-old male patient presented to the emergency department with one month of fatigue and five days of progressive dyspnoea and fever. Three months earlier he had been diagnosed with idiopathic hypereosinophilia. Extensive diagnostic evaluation at that time revealed neither a cause for secondary eosinophilia nor any cardiac abnormalities. Oral prednisone was given for two months, but was gradually tapered since there was no decline in eosinophil count.

**Figure 1:**
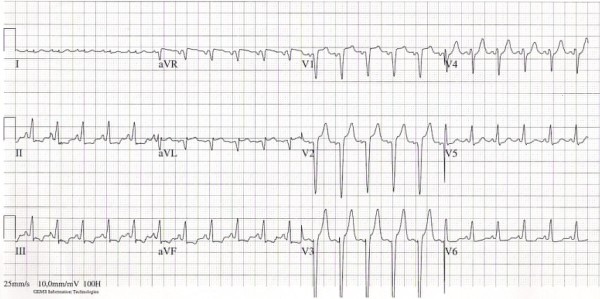
Electrocardiogram at admission showing a sinusrythm with ST-elevations in V1-V3 and ST-depressions in II, III, AVF, V5 and V6.

**Figure 2:**
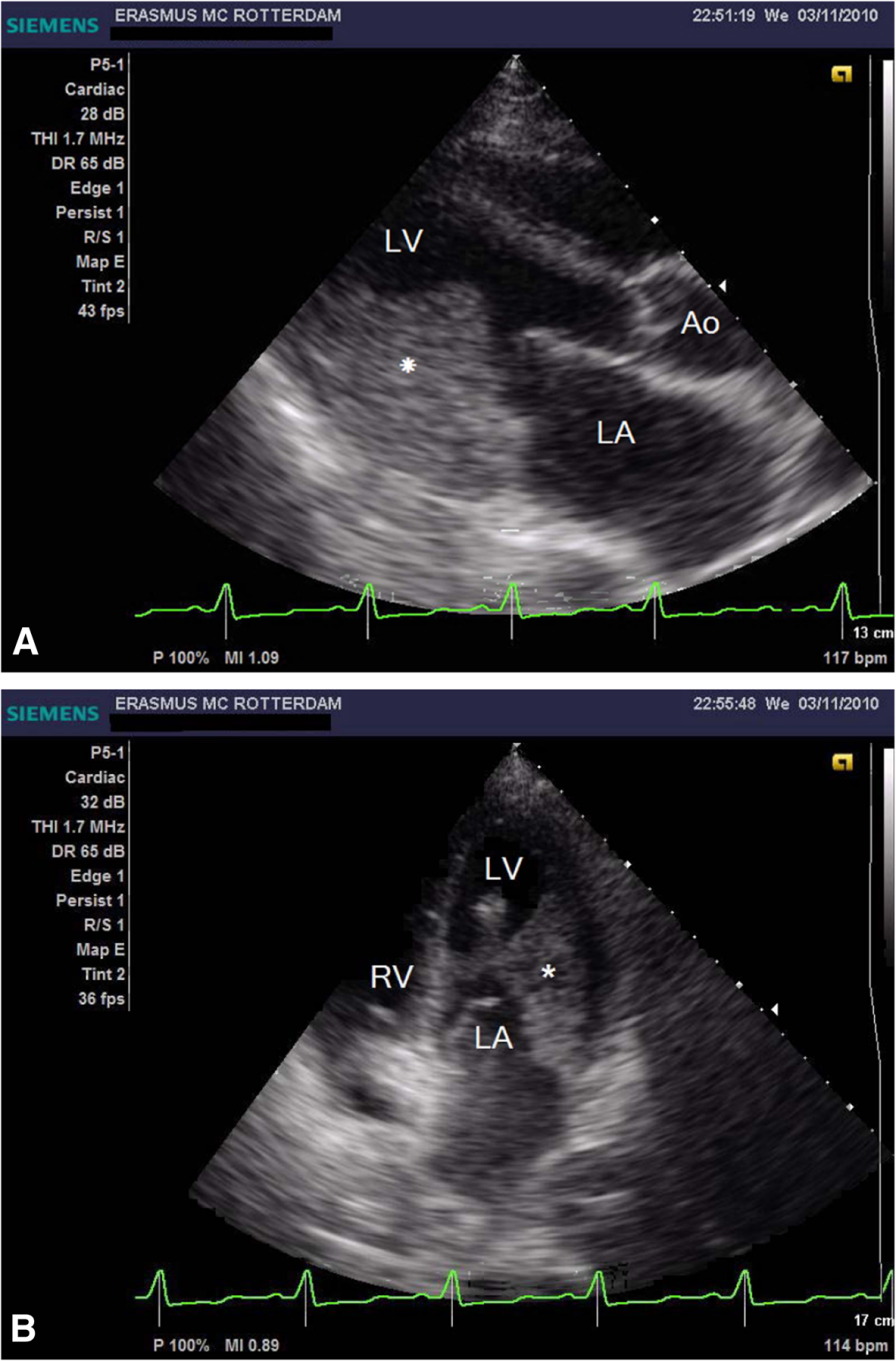
A and B, Echocardiographic parasternal long axis view and four-chamber view at presentation, showing big thrombotic mass with the base at the posterior wall and infiltration of the left ventricular wall.

On admission we saw an acutely ill-looking man, temperature 38.1°C, blood pressure 80/47 mmHg, heart rate 137 beats/min and a saturation of 90% with 15 liters per minute of oxygen therapy by a non-rebreathing mask. Auscultation of the heart revealed no murmurs; diffuse inspiratory crackles were heard during ausculation of the lungs. No other abnormalities were found. Laboratory evaluation revealed a white blood cell count of 96.8 × 10^9^/L, of which 84.2 × 10^9^/l were eosinophils (87%), hemoglobin 8.6 mmol/l, thrombocytes 238 × 10^9^/L, C-reactive protein 122 mg/L, creatinine 141 umol/L, lactate dehydrogenase 426 U/L, aspartate aminotransferase 28 U/L and creatine phosphokinase 45 U/L. Arterial blood gas analysis showed a pH of 7.46, pCO2 4.3 kPa, pO2 8.5 kPa and bicarbonate 23 mmol/L. Chest X-ray showed bilateral pulmonary congestion. The electrocardiogram showed a sinus tachycardia, a QS complex in V1 and V2, 2 mm. ST-elevations in V1-V3 and ST-depressions in II, III, AVF, V5 and V6 (Figure 1). Transthoracic echocardiography revealed a large mass in the left ventricle (LV) attached to the posterolateral wall with severe inflow- and outflow tract obstruction; echo Doppler maximal LV outflow tract velocity was 6 m/s (Figure 2A and B). The posterior mitral valve leaflet (PMVL) was completely embedded in the mass. The left and right ventricular function was however fully normal. Because of progressive respiratory failure, the trachea was intubated and mechanical ventilation was initiated. A CT-scan of the thorax confirmed the large (thrombus) mass in the left ventricle, left and right atrial dilatation, pulmonary edema and bilateral pleural effusions. Cardiac magnetic resonance imaging (MRI) showed endomyocardial fibrosis and necrosis and a large thrombus in the left ventricle with obstruction of the left ventricle outflow tract. A diagnosis of Löffler endocarditis was made. Pharmacological therapy was initiated with diuretics, high dose beta-blockers, methylprednisolone (1000 mg a day for three days) and anti-coagulation along with antiplatelet agents acetylsalicylic acid and clopidogrel. Mechanical ventilation was necessary for twelve days. After this period weaning and extubation was successfully. Because of severe diastolic dysfunction with an inflow and outflow tract obstruction of the left ventricle due to the thrombus, inotropics were not helpful and patient received only on the first day norepinephrine. Initially there was a marked decline in his eosinophil count (white blood cell count of 32 × 10^9^/L with 17 × 10^9^/L eosinophils (53%) however this was of short duration. Therefore we started therapy with interferon alpha and hydroxycarbamide resulting in a gradual decline of the eosinophil count (white blood cell count of 6.6 × 10^9^/L, of which 2.28 × 10^9^/L (34.5%) were eosinophils). Signs of bone marrow depression with anemia and thrombocytopenia complicated this therapy. Interferon alpha therefore was stopped.

**Figure 3:**
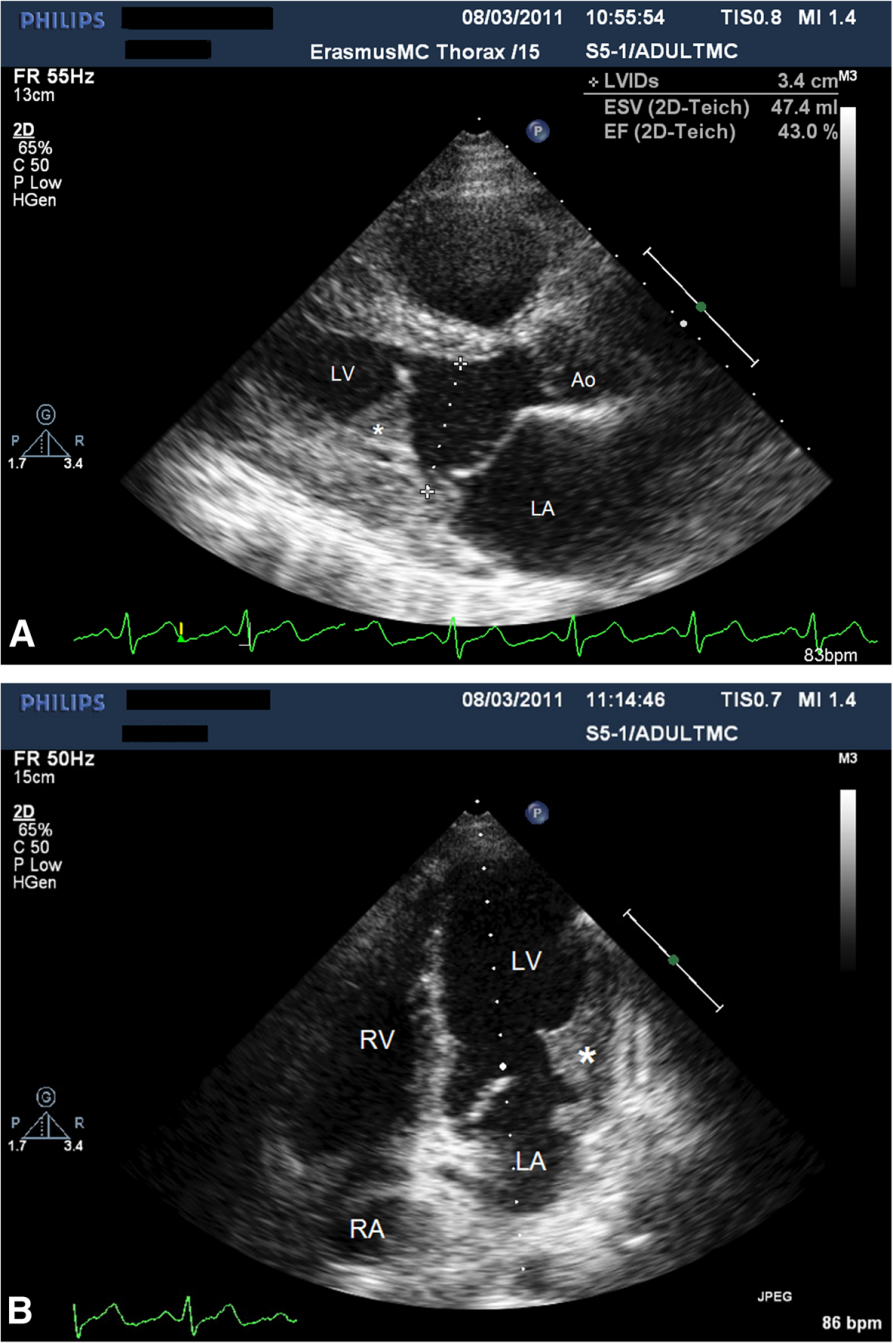
A and B, Same view at 4 months of follow-up, demonstrating smaller but evidently present thrombotic mass, with embedded posterior leaflet apparatus of the mitral valve, resulting in severe mitral regurgitation (not shown).

Two months later the cardiac MRI was repeated and still showed the large mass in the left ventricle but with a 25% decline. During follow-up at four months there was persistent heart failure, New York Heart Association Class III to IV. Echocardiography showed also a severe mitral regurgitation because the posterior mitral valve leaflet was embedded in the thrombus mass (Figure 3A and B) and a severe secondary pulmonary hypertension (estimated pulmonary artery systolic pressure 60 to 65 mm Hg). In a multidisciplinary team discussion we concluded that mitral valve replacement was the only option for this young patient with severe heart failure. A surgical correction of the mitral regurgitation was performed through sternotomy. A mitral valve bioprosthesis was inserted. After this operation the patient recuperated well, although the eosinophilia persisted (white blood cell count of 12 × 10^9^/L, of which 85% were eosinophils). Therefore, interferon alpha was added again to the therapy with hydroxycarbamide. However, high doses were needed and patient developed again a pancytopenia. Additional treatment with Imatinib (a tyrosine kinase inhibitor) and Mepolizumab (a humanized monoclonal antibody against interleukin-5) did not lead to a satisfactory further decrease in eosinophil count. Eventually it was decided to perform an allogenic stem cell transplantation. Apart from a mild to moderate graft versus host reaction (GVHD), this procedure was uncomplicated. Currently, patient is doing well. After transplantation his eosinophilic count is 1.24 × 10^9^/L (20%). As post transplantation chimerism analysis showed more than 95% donor cells, the remaining eosinophilia is considered to be due to the GVHD.

## Discussion

Acquired eosinophilia can be classified into secondary (cytokine-driven reactive phenomenon), clonal (presence of a bone marrow histological, cytogenetic or molecular marker of a myeloid malignancy) and idiopathic (neither secondary nor clonal) categories [2]. In 1975 Chusid et al. described diagnostic criteria for idiopathic HES that remain in use today. These are blood eosinophilia of >1500 cells per microliter for more than six consecutive months, absence of an underlying cause of hypereosinophilia despite extensive diagnostic evaluation, and organ damage or dysfunction as a result of local release of the toxic contents of eosinophils [1]. HES affects mostly men between 20 and 50 years of age, with a peak in the 4^th^ decade of life [3,4]. There are no published data regarding its incidence/prevalence, although the syndrome is considered rare in adults and very rare in children.

The exact etiology of HES is unknown; however, the process probably involves a dysregulation of IL-3, IL-5 and/or GM-CSF, which are all eosinophil growth factors [3,5].

Clinical manifestations of HES are markedly heterogeneous as the disease can either be completely asymptomatic or involve multiple organs. In essence, any organ is vulnerable to eosinophilia-associated tissue damage. Thromboembolic disease involving the cardiac chambers and/or both the venous and arterial vessels is not infrequent [2]. Cardiac involvement occurs in more than 75% of patients with HES and is the major cause of morbidity and mortality [6]. Cardiac involvement in HES usually follows three stages. The first is an acute necrotic stage. This stage is usually neither recognized clinically nor diagnosed, like in our case. In this stage there is damage to the endocardium and infiltration of the myocardium with eosinophils and lymphocytes with histopathological evidence of myocardial necrosis and eosinophil degranulation and eosinophil micro abscesses [3,7]. The second stage concerns thrombosis with formation of thrombi along the damaged endocardium of either one or both ventricles and occasionally in the atrium. Outflow tracts near the aortic and pulmonic valves are usually spared, although rarely thrombus may involve these valves and thrombus may form on atrioventricular valve leaflets [3,8-10]. This stage is found in those with a mean 10-month duration of eosinophilia [3]. In the third late fibrotic stage progressive scarring may lead to entrapment of chordae tendineae resulting in mitral and/or tricuspid valve regurgitation and endomyocardial fibrosis producing a restrictive cardiomyopathy [3].

To diagnose HES and Löffler endocarditis, echocardiography enables sufficient detection of thickened endocardium and intraventricular thrombus. Nevertheless, acute necrotic stage can come along with absolute normal echocardiographic findings [3]. Endomyocardial thickening is seen in 68% of patients on echocardiography and is progressive [11]. Apical thrombus in the presence of normal apical contraction is one of the main clues in suspected Löffler’s endomyocarditis [12]. There are three primary goals for the management of HES: 1) reduction of peripheral and tissue levels of eosinophils; 2) prevention of end-organ damage; and 3) prevention of thrombo-embolic events in patients at risk [13,14]. Patients without progressive organ system dysfunction typically do not require specific treatment, however, these patients should be monitored closely [14]. Corticosteroids were initially the mainstay of HES treatment and are currently recommended as first-line therapy [15,16]. Cytotoxic agents like hydroxyurea are indicated in HES subsets with corticosteroid resistance or when corticosteroid tapering is necessary [16]. Interferon-alpha is an immunomodulator that reduces Th2-mediated IL-5 production, synthesis of GM-CSF and release of eosinophil-specific neurotoxin and eosinophil cationic protein and indirectly inhibits eosinophil differentiation [5,13,17]. It is recommended for use in HES patients with organ damage and corticosteroid/cytotoxic treatment failure [17,18]. Allogenic stem cell transplantation has also been reported to be a potentially curative therapy. It is the ultimate therapeutic measure in case of therapeutic refractoriness or intolerance to available therapies or in patients who present with progressive life-threatening end-organ damage. The optimal preparative regime is not well established and it is associated with major morbidity and even mortality [19,20]. Several therapies are currently under investigation as potential HES therapies, like Mepolizumab, a fully humanized monoclonal IgG anti-IL5 antibody. The secondary treatment should be directed at cardiac complications, e.g., heart failure and the presence of intracardial thrombus. In the view of subclinical progression of the cardiac involvement, especially in the primary stage of HES, our case suggests very early and aggressive anticoagulation, regardless of the initial (negative) cardiac evaluation. Occasionally, surgical therapies are needed for cardiovascular complications. Surgical experience of patients with valvular dysfunction secondary to HES is limited [21]. Valve replacement is most often performed but the choice between a mechanical or biological prosthesis in this setting poses a difficult problem. Mechanical valves have a high incidence of recurrent obstructive thrombosis and therefor a bioprosthesis is recommended despite associated restrictive cardiomyopathy [6].

## Conclusions

Hypereosinophilic syndrome is a rare cause of cardiac failure resulting in cardiogenic shock due to endomyocardial thickening and thrombus formation. Both may impede left ventricular in- and outflow tract. The risk increases when the duration of hypereosinophilia is more than ten months. Besides first line therapy (corticosteroids), early aggressive anticoagulation therapy is warranted. In more severe cases acute surgical intervention is necessary.

## Consent

Written informed consent was obtained from the patient for publication of this report and any accompying images.

## Abbreviations

LV: Left ventricle; LA: Left atrium; Ao: Aorta; RV: Right ventricle; RA: Right atrium; *: Thrombus mass.

## Competing interests

The authors declare that they have no competing interests.

## Authors´ contributions

All the authors participated in the treatment of this patient. ND Niemeijer drafted the manuscript. PLA van Daele and NJM van der Meer helped to draft the manuscript and also revised it critically. OJL Loosveld, K Caliskan and F Oei have been involved in revising it critically. Kadir Caliskan did made the echocardiography and provided the figures. F Oei did the mitral valve replacement operation. All authors read and approved the final manuscript.
